# Effect of Bow Camber and Mass Distribution on Violinists' Preferences and Performance

**DOI:** 10.3389/fpsyg.2021.769831

**Published:** 2021-11-03

**Authors:** Aurélie Tomezzoli, Benjamin Michaud, Eric Gagné, Mickaël Begon, Sonia Duprey

**Affiliations:** ^1^Univ Lyon, Université Claude Bernard Lyon 1, Univ Gustave Eiffel, LBMC UMR_T9406, Lyon, France; ^2^Laboratoire de Simulation et de Modélisation du Mouvement, École de Kinésiologie et des Sciences de l'Activité Physique, Faculté de Médecine, Université de Montréal, Montréal, QC, Canada; ^3^Wilder & Davis, Montréal, QC, Canada; ^4^Research Center, Sainte-Justine Hospital, Montréal, QC, Canada

**Keywords:** violin, bow, preference, musical analysis, acoustic

## Abstract

Little is known about how bow mechanical characteristics objectively and quantitatively influence violinists' preferences and performance. Hypothesizing that the bow shape (i.e., camber) and mass distribution modifications would alter both violinists' appreciations of a bow and objective assessments of their performance, we recruited 10 professional violinists to play their own violin using 18 versions of a single bow, modified by combining three cambers and six mass distributions, in random order. A musical phrase, composed for this study, was played legato and spiccato at three octaves and two tempi. Each violinist scored all 18 bows. Then, experts assessed the recorded performances according to criteria inspired by basic musical analysis. Finally, 12 audio-descriptors were calculated on the same note from each trial, to objectivise potential acoustic differences. Statistical analysis (ANOVA) reveals that bow camber impacted the violinists' appreciations (*p* < 0.05), and that heavier bow tips gave lower scores for spiccato playing (*p* < 0.05). The expert evaluations reveal that playing with a lighter bow (tip or frog), or with a bow whose camber's maximum curvature is close to the frog, had a positive impact on some violinists' performance (NS to *p* < 0.001). The “camber-participant” interaction had significant effects on the violinists' appreciations (*p* < 0.01 to *p* < 0.001), on the expert's evaluation and on almost all the audio-descriptors (NS to *p* < 0.001). While trends were identified, multiple camber-participant interactions suggest that bow makers should provide a variety of cambers to satisfy different violinists.

## Introduction

Violin bows' shape and materials have changed little since the end of the eighteenth century. However, trade restrictions have made the wood used in bow sticks, Pernambuco (*Paubrasilia echinata*, also termed brazilwood and derived mainly from Brazilian forests), less available and more expensive (Convention on International Trade in Endangered Species of Wild Fauna Flora, 2017). This increasing rarity raises the question of what makes a “good” pernambuco bow, if satisfactory alternatives are to be developed: both the point of view of violinists, through the assessment of their preferences, and of listeners, through the assessment of violinists' musical performance should be considered.

The scientific interest for string vibrations is ancient, as links between a monochord vibration and sound pitches are well known since the Antiquity (Abromont and de Montalembert, 2001), and is still relevant, as can be seen for instance with the studies of the non-steady parts of bowing, i.e., the attack and release of the sound also known as transients (Badeau, 2005; Castellengo et al., 2015). In further work, the relationships between the string vibration and the bow have been studied (Schoonderwaldt et al., 2007; Schoonderwaldt and Altenmüller, 2009). With the development of new technological possibilities appeared a growing interest for the upper limb biomechanics during the bowing (Schoonderwaldt and Altenmüller, 2011; Kelleher et al., 2013), as well as the description of the motion of the bow itself, which remains a technological challenge (Provenzale et al., 2021).

Most studies investigating violinists' preferences have focused on the violin itself (Saitis et al., 2012, 2015; Fritz and Dubois, 2015). Instrumentalists' rankings of violins have been shown to be reproducible (Saitis et al., 2012), and the more precise the musical task, the more the instrumentalists agree on their assessments of a violin. Violinists also discriminate better between violins, in terms of richness of sound, when playing rather than listening to them (Saitis et al., 2015). Such studies set the example in experimental design to assess instrumentalists' preferences. However, no study has addressed violin bow preferences. A single study (Caussé et al., 2001) explored potential connections between verbal descriptors used by instrumentalists and the static (measured) or dynamic (estimated by finite element modeling) mechanical properties of the bow. Their preliminary findings showed no evidence of a link. Other studies on the violin bow addressed its modal and acoustical responses (Ravina et al., 2008) and its spectral content (Schoonderwaldt and Altenmüller, 2009), but without considering how these might relate to violinists' preferences (Woodhouse, 2014). There is clearly a gap in our knowledge concerning the relationship between bow mechanical characteristics and preferences.

Determining whether a musical performance is satisfactory inevitably involves human perception. Although human evaluations are subjective by nature—i.e. dependent on personal musical culture, place and time—their reproducibility has been documented: music student grades have been reported to show an intra-rater correlation coefficient of 0.87–0.99 (Franzén, 1969; Beazley, 1981) and an inter-rater correlation of 0.95–0.95 (Beazley, 1981). Evaluations by experts were found to be most reproducible if the judges were experienced and judged from recordings rather than from real-time performances (Salvador, 2010). Furthermore, musicians were found to be more accurate than non-musicians in their perceptions (McAdams et al., 1999; Fritz et al., 2008).

A complementary approach to performance rating involves calculating audio-descriptors, which are numerical indicators that account for particular dimensions of a sound. The calculation is based either on sound energy envelopes or on frequency spectrums, using either the Fast Fourier Transform (FFT) or the harmonic model (Table 1). Some parameters are temporal, describing the variation of one parameter across time, while others are global, describing an entire sound (Peeters et al., 2011). Audio-descriptors are used in several ways in the literature. The approach taken by music information research, for example to automatically classify instruments, distinguishes between different sounds by including as many descriptors as possible, even those not relevant in terms of human perception. This approach has been found effective in this context, correctly classifying more than 90% of the instrumental sounds tested (Siedenburg et al., 2016). Another approach, used in musical psychology, assesses the dissimilarity between sounds using a small number of audio-descriptors whose relevance in terms of human perception has been shown (Table 1; Peeters et al., 2011). For the violin, research has focused on the correlation of audio-descriptors with verbal descriptors (Fritz et al., 2012; Saitis et al., 2012). As for bow assessment based on audio-descriptors, the impact of different bowing parameters, as speed and pressure, has been assessed, but without examining bow mechanical characteristics themselves. Schoonderwaldt and Altenmüller (2009) showed that the spectral centroid (Table 1) increases mainly with the pressure exerted by the bow, especially when played close to the bridge. Moreover, the fundamental frequency of the note decreases when bow pressure is high, especially when played far from the bridge. Conversely, fundamental frequency increases when a string is played rapidly near the bridge (Schoonderwaldt and Altenmüller, 2009). To date, audio-descriptors have not been used to assess the sound modifications generated by different bows.

**Table 1:** Main audio-descriptors as calculated in Peeters et al. (2011), variables transformed in our study, and camber-participant interaction effect on audio-descriptors.

**Sound representation**	**Audio-descriptor**	**Definition**	**Variable transformation**	**Camber—subject interactions**
Temporal representation	RMS envelope	RMS of the amplitude of the temporal energy	ln (*x*)	*p* < 0.001
	Attack	Attack duration	1/x	*p* < 0.01
	Release	Duration of the last phase of sound	$\sqrt{\left(1.55+\text{ln }\left(\text{x}\right)\right)}$	*p* < 0.001
FFT representation	Spectral centroid	$\mu_{1}\left(t_{\text{m}}\right)=\;\overset{K}{\underset{k=1}{\sum }}f_{k}\;\cdot \;p_{k}\left(t_{\text{m}}\right)$ where $p_{k}\left(t_{\text{m}}\right)=\left[a_{k}\left(t_{\text{m}}\right)\right]\;/\;\overset{K}{\underset{k=1}{\sum }}a_{k}\left(t_{\text{m}}\right)$	x	*p* < 0.001
	Spectral variation (i.e., spectral flux)	$variation\;\left(t_{\text{m}},\;t_{\text{m}-1}\right)=1-\;\frac{\overset{K}{\underset{k=1}{\sum }}a_{k}\left(t_{\text{m}-1}\right)a_{k}\left(t_{\text{m}}\right)}{\sqrt{\overset{K}{\underset{k=1}{\sum }}{a_{k}\left(t_{\text{m}-1}\right)}^{2}}\sqrt{\overset{K}{\underset{k=1}{\sum }}a_{k}{\left(t_{\text{m}}\right)}^{2}}}$	*x*	*p* < 0.05
Harmonic representation	Fundamental frequency	F_0_	*x*	NS
	Inharmonicity	$inharm\left(t_{\text{m}}\right)=\;\frac{2}{f_{0}\left(t_{\text{m}}\right)}\;\frac{\overset{H}{\underset{h=1}{\sum }}\left(f_{\text{h}}\left(t_{\text{m}}\right)-hf_{0}\left(t_{\text{m}}\right)\right)\;a_{\text{h}}^{2}\left(t_{\text{m}}\right)}{\overset{H}{\underset{h=1}{\sum }}a_{\text{h}}^{2}\left(t_{\text{m}}\right)}$	ln (*x*)	*p* < 0.05
	Noisiness	$noisiness\left(t_{\text{m}}\right)=\;\frac{E_{\text{N}}\left(t_{\text{m}}\right)}{E_{\text{T}}\left(t_{\text{m}}\right)}$ with *E*_N_(*t*_m_) = *E*_T_(*t*_m_)− *E*_H_(*t*_m_); $E_{\text{T}}\left(t_{\text{m}}\right)=\;\underset{k}{\sum }a_{\text{k}}^{2}\left(t_{\text{m}}\right)$	ln (1 – *x*)	*p* < 0.001
	Odd-to-even harmonic ratio	$OER\left(t_{\text{m}}\right)=\;\frac{\overset{H/2}{\underset{h=1}{\sum }}\;a_{2\text{h}-1}^{2}\left(t_{\text{m}}\right)}{\overset{H/2}{\underset{h=1}{\sum }}a_{2\text{h}}^{2}\left(t_{\text{m}}\right)}$	ln (*x*)	*p* < 0.01
	Tristimuli (x3)	$T_{1}\left(t_{\text{m}}\right)=\;\frac{a_{1}\left(t_{\text{m}}\right)}{\overset{H}{\underset{h=1}{\sum }}a_{\text{h}}\left(t_{\text{m}}\right)}$	ln (*x*)	NS
		$T_{2}\left(t_{\text{m}}\right)=\;\frac{a_{2}\left(t_{\text{m}}\right)\;+\;a_{3}\left(t_{\text{m}}\right)+\;a_{4}\left(t_{\text{m}}\right)}{\overset{H}{\underset{h=1}{\sum }}a_{\text{h}}\left(t_{\text{m}}\right)}$	*x*	NS
		$T_{3}\left(t_{\text{m}}\right)=\;\frac{\overset{H}{\underset{h=5}{\sum }}a_{\text{h}}\left(t_{\text{m}}\right)}{\overset{H}{\underset{h=1}{\sum }}a_{\text{h}}\left(t_{\text{m}}\right)}$	*x*	*p* < 0.05

*f_k_, frequency of the partial k; p_k_, normalized amplitude; f_0_, fundamental frequency; h, multiple of the fundamental frequency; a_h_(t_n_), f_h_(t_n_), Φ_h,0_(t_n_), amplitude, frequency and initial phase; E_T_ (t_m_), total spectral energy; E_N_ (t_m_), noise energy*.

The main objective of this study was to investigate the influence of bow mechanical characteristics, specifically its camber and mass distribution, on instrumentalists' preferences and on their musical performance. In keeping with the above literature, we chose to evaluate musical performance on the basis of both expert assessment and audio-descriptors.

## Method

### Data Collection

Ten professional musicians (9 women and 1 man, aged 27.7 ± 7.6 years), who had been playing the violin for 21.0 ± 8.5 years were recruited to participate in this study. The protocol was approved by the ethics committee of the University of Montreal (17-018-CERES-D). The violinists played their own personal violin, while a single bow (mass: 62 g; natural camber, i.e., bow shape: camber 1) was used, modified into 18 camber/mass distribution configurations.

A bow maker (coauthor EG) modified the bow behind a curtain, changing its camber and adding mass randomly in two steps [see the Supplementary Material and the video (S2M Lab, 2017) minute 0:42]. First, one of the three cambers was randomly selected. After being heated (see the Supplementary Material), the bow was bent to match one of three defined patterns. Then masses were randomly added at the tip (0, 1 or 2 g, i.e., 1.6 or 3.2% of the bow weight) and/or at the frog (0 or 2 g), using sticky gums. All camber and mass combinations were tested, i.e., 18 bow conditions for each violinist, except for one of the cambers (camber 1) for one of the violinists, due to lack of time. For 6 out of the 10 participants, one of the 18 conditions, randomly chosen for each camber, was repeated in immediate succession. The bow maker pretended to modify the bow as usual, ensuring that the violinist did not know the two conditions were identical.

**Figure 1:**
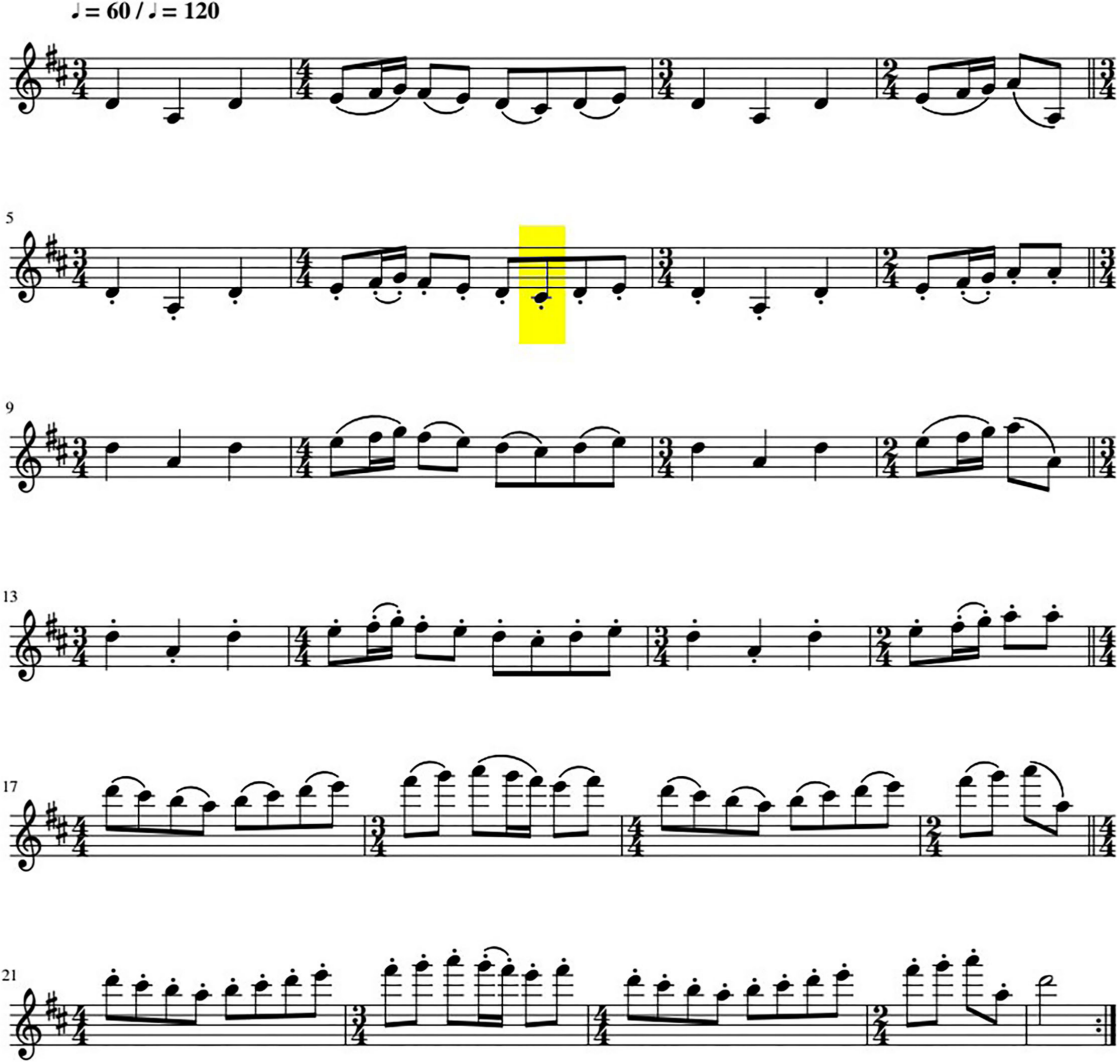
The piece of music, consisting of two sections (spiccato, then legato), both played at three octaves. The C# analyzed using audio-descriptors is highlighted in yellow.

The piece of music (Figure 1), especially composed to explore different facets of the bowing technique while keeping a certain degree of ecological validity, consisted of two sections, each involving a different type of articulation: first legato, then spiccato. Both were played over three octaves. The entire piece of music was played at two tempi, 60 beats per minute or 120 (an electronic metronome was used), in random order, for a total of 12 musical sections. The sound was recorded with a Zoom Q3HD recorder (condenser microphone, XY, 120° angle), set at a sampling rate of 44.1 kHz. The recorder was placed on the lectern, facing the violinist. The lectern position relative to the performer was kept relatively constant during recordings by floor markings.

### Evaluation

After each trial, the participants gave their overall appreciation of the bow, rating it from 0 to 10, as well as an appreciation specifically for playing legato and spiccato. No instructions were given regarding rating criteria.

From the recordings, two-step randomized by violinist and then by audio file, a first expert evaluated musical performance (coauthor TA, a composer). She searched for four types of defects, using a method inspired by musical analysis techniques: timbre errors (squeaking, wrong string played, unexpected bounced bowstroke), lack of pitch accuracy, lack of rhythm precision and articulation errors. She defined the defects in writing and via sound samples. Penalty scores (timbre score, pitch score, rhythm score and articulation score) were calculated for each bow configuration for each participant by incrementing them (+1) whenever a musical section contained at least one of the targeted defects. An overall penalty score was also calculated for the whole piece of music and for all the sections related to each musical parameter (types of articulation, octave and tempo). Penalty scores were then fitted to a scale ranging from 0 (flawless performance) to 10 (all musical sections were flawed). Then, 30 audio files (three per participant) were randomly selected. They were assessed again 1 month later by the same expert and a second expert (coauthor MB, a violinist and the composer of this piece of music) to assess grading repeatability, using coefficients of variation.

After averaging the two voices of the stereo recording, audio-descriptors that seemed the most relevant in terms of human perception were calculated using Matlab's Timbre Toolbox (Peeters et al., 2011). To avoid redundancy, we choose the descriptors which were the less linked to each other, i.e., that had a median rank correlation lower than 0.8 in Peeters et al. (2011) (see Table 1). As recommended by Peeters et al. (2011), the temporal audio-descriptors were characterized by their median value. Audio-descriptors were calculated on the C#3 (expected frequency of 277 Hz) spiccato tempo 60 (see Figure 1). This note was manually extracted using Audacity (Audacity Team, 2018).

### Statistics

To assess the repeatability of violinists' preferences, differences between their assessments of the two identical bow trials were calculated. Then, after checking the normality of these differences, the coefficient of variation between the first and second trial was calculated. The coefficient of determination (*R*^2^) of an analyse of variance (ANOVA) was used to assess the percentage of variance in preferences due to the difference between two repeated tests.

The influence of bow characteristics was assessed using the bow cambers, the mass added at the tip and the mass added at the frog as explanatory variables. Dependent variables were the violinists' preferences, the expert's penalty score and the audio-descriptors. The distribution of each variable was checked graphically to ensure its normality and perform any necessary transformations (Table 1). Homoscedasticity among subgroups was tested using a Levene test. After verification of these conditions of applicability, one-factor ANOVAs were performed. Due to high inter-participant variability, the factors were all re-tested with a two-factor ANOVA including a participant effect. More complex models were developed using this approach, i.e., by progressively adding the most significant variables (bottom-up approach).

Pearson's linear correlation coefficients were calculated among the violinists' different appreciations (overall, legato and spiccato playing) and among penalty scores and audio-descriptors, after normalizing each parameter by violinist (same mean and standard deviation for each) and graphically verifying the shape of correlations on scatter plots.

## Results

### Violinists' Preferences

**Figure 2:**
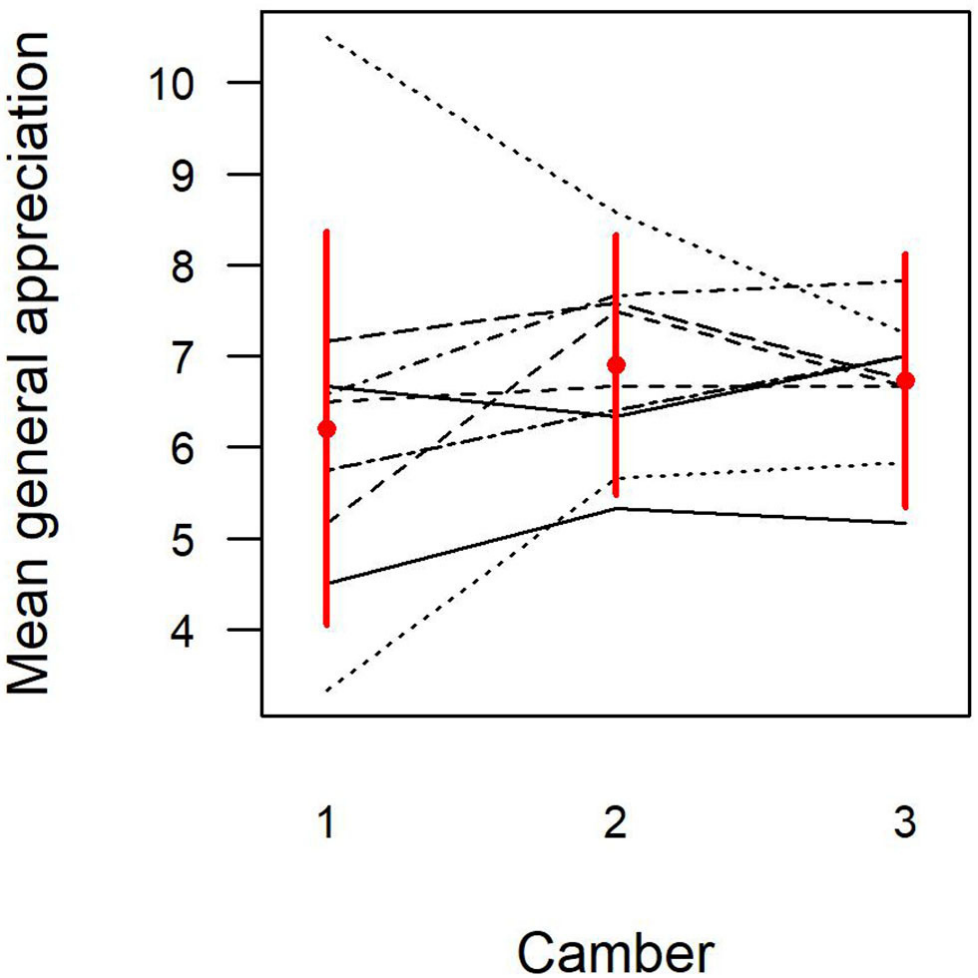
Violinists' bow appreciations—average overall appreciation according to camber (1, 2 or 3) and violinist. Lines connect the values for each individual violinist.

The mass at the tip had a significant effect on appreciations of spiccato playing, and the camber had a significant effect on all appreciations. Since there was a major participant effect (*R*^2^ = 0.31–0.37, *p* < 0.001, vs. *R*^2^ = 0.2 at most, not significant, for the explanatory variables), it was taken into account simultaneously through two-factor ANOVA. Specifically, added mass at the tip of the bow reduced ratings for the spiccato on average (5.8, 6.5 and 6.6/10, for 2, 1 and 0 g addition, respectively, *p* < 0.05). Camber 1 was always the lowest rated (overall ratings of 6.2, 6.9 and 6.7/10, for cambers 1, 2 and 3, respectively), except by one violinist. However, there were differences in effect size and in preferences between cambers 2 and 3, generally explained more by a camber-participant interaction (*p* < 0.01 to *p* < 0.001) than by the camber itself (*p* < 0.05) (Figure 2). In the three-factor model, there was no interaction between violinists, added mass and camber.

**Figure 3:**
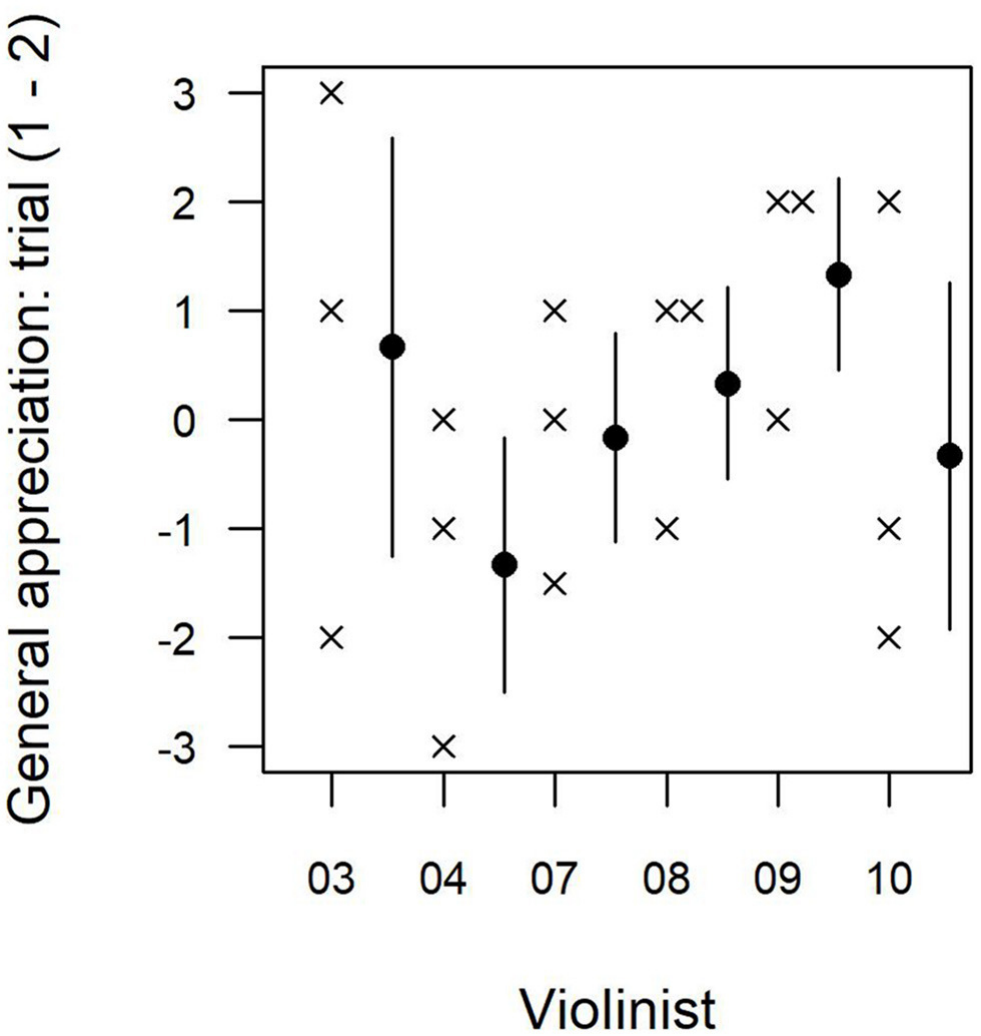
Differences in violinists' general appreciations for the two tests performed under identical bow conditions (trial 1–trial 2) for six participants (3, 4 and 7–10).

In terms of repeatability of the violinists' appreciations, the mean of the absolute value of the difference between the two identical bow trials was 1.4/10 for the overall and legato assessments and 1.6/10 for the spiccato assessment. Half the participants raised their rating on average in the second trial, while the other half lowered their rating (Figure 3). The coefficient of variation between the values given for the first and second trials was 0.26 for overall assessment, 0.33 for spiccato, 0.27 for legato. The percentage of variance explained by difference between the two identical bow trials was 23% for the overall assessment, 30% for the legato assessment and 17% for the spiccato assessment.

After normalization by violinist, links between overall ranking and appreciations for spiccato and legato playing were respectively *r* = 0.84 and 0.87. Correlation between the latter two appreciations was *r* = 0.62.

### Experts' Penalty Ratings

The rating agreement between evaluations 1 month apart was very good (81.4%), indicating low intra-expert variability. The consistency of ratings between experts was good (70.1%), indicating reasonable inter-expert variability. Once again, all penalty ratings showed a major participant effect (*R*^2^ = 0.34–0.78, *p* < 0.001, vs. at most R^2^ = 0.2, NS, for the explanatory variables). After taking into account the participant effect, the main results were that a mass of 0 g at the tip had a positive effect on timbre ratings (5.6 vs. 6.3 and 6.0/10 for a 1 and 2 g mass, respectively, *p* < 0.05), and that a mass of 0 g at the frog had a positive effect on playing at high pitches (two last lines of the piece of music, Figure 1), depending on the violinist (mean: 4.1 vs. 4.2/10, *p* < 0.05).

Camber had an influence at low pitches and in fast passages (*p* < 0.05), in both of which cases camber 3 obtained the lowest penalty ratings (mean: 3.2 vs. 3.5 and 3.6/10 for camber 3, 1 and 2, respectively at low pitch and 3.4 vs. 3.8 and 3.7/10, respectively in fast passages). There were camber-participant interactions for rhythmic precision, for respect of articulation, at middle-range pitches (*p* < 0.05) and for overall ratings (*p* < 0.001).

After normalization by violinist, no links between timbre, pitch, rhythm and articulation penalty scores were found, nor between mutually exclusive musical parameter penalty scores (for example, between penalty scores for each of the three octaves).

### Audio-Descriptors

For the audio-descriptors that did not fit a normal distribution, variables were transformed to ensure normality (Table 1). For each audio-descriptor, there was a major participant effect (*R*^2^ = 0.18–0.78, *p* < 0.001, vs. at most a non-significant *R*^2^ = 0.2 for the explanatory variables). When the participant effect was taken into account, camber-participant interaction had a statistically significant effect on almost all audio-descriptors, whereas mass additions had no effect (Table 1).

After normalization by violinist, multiple links were found between audio-descriptors, computed two by two.

## Discussion

The main objective of this study was to investigate the influence of bow mechanical characteristics on both instrumentalists' preferences and their musical performance. As hypothesized, bow modifications altered the violinists' appreciations, especially camber changes. Concerning musical performance, significant “camber-participant” interactions were found for each audio-descriptor. The experts' evaluations revealed that a camber with maximum curvature closest to the frog (Camber 3) had positive effects on playing, while added mass at the tip or frog had negative effects.

Acoustic response can differ across bows (Ravina et al., 2008). Specifically, we found that camber has a major impact, much more pronounced than bow mass distribution, on many sound parameters and on violinists' appreciations. The camber with maximum curvature closest to the tip (Camber 1) scored lowest, except with one violinist. Differences in violinists' preferences regarding the two other cambers, i.e., camber-participant interaction, could be explained by differences in the types of bow they habitually use, the ones with cambers most similar to theirs being possibly preferred. Documenting the characteristics of the bows generally used by the instrumentalists involved in experimental studies might therefore be worthwhile. On the other hand, these differing preferences could also reflect musicians' personal aesthetic aims. Actually, overall quality rating and preference rating are known to be poorly correlated (Wollman et al., 2014), likely suggesting different expectations regarding musical performance. In the end, since our violinists' appreciations and experts' evaluations often depended on the violinist (camber-participant interaction), a good general principle for bow makers would be to provide bows with different cambers (affecting overall bow stick design) to satisfy diverse violinists.

Throughout the history of bow making, a balance has been sought between bow length, mass and “nervosity,” i.e., its ability to respond quickly to the violinist gesture (Bachmann et al., 2001), which seems likely to refer to its dynamic properties. In the present study, the mass added at the tip and frog affected both the violinists' appreciations of the bow and their performance. At the tip, added mass had a negative impact both on instrumentalists' ratings for spiccato playing and on the frequency of timbre flaws as evaluated by the expert. A deleterious effect on the spiccato was expected: the bow “bounces” less well with more weight at the tip, since the center of mass may have shifted and/or the bow may be more difficult to control. Adding 2 g to the frog also had a negative impact, i.e., higher expert penalty ratings, with some players producing more defects in the treble. Bow mass and nervosity are linked to the nature of the materials used and to the geometry of the bow (Bachmann et al., 2001). Our data, together with the new prospect of using carbon fibers to make lighter bows, encourage reconsideration of the total weight of bows, as well as their mass balance and their dynamic behavior. This field of research is complex, as the bow material and geometry also impact the risk for bow fatigue failure (Bachmann et al., 2001). The expectations of different musicians have to be accounted, as highlighted above, and the musical patterns used for assessing the bow quality can also be adjusted to specific musical repertoires (Koechlin, 1956; Penesco, 1986).

While the present protocol was designed in a comprehensive way, taking into account instrumentalists' preferences, experts' evaluations and effects on sound, some limitations remain. In terms of expert evaluation, the reproducibility, even though it is reasonable (consistency of 81.4 and 70.1% for intra- and inter-expert assessments), could be improved. As for audio-descriptors, calculations could be extended to include more musical notes (Tomezzoli et al., 2019). Furthermore, given the multiple links found between the audio-descriptors used here, their number could be reduced. Finally, the highly significant inter-participant effect found here reflects not only the effect of inter-individual variations between participants but also differences in the violin and sound recording. Studies with larger samples of instrumentalists will need to be performed before these findings can be generalized to the entire population of violinists.

## Author's Note

The results reported in the present article were partly presented at the 44th congress of the French Biomechanics Society (see Tomezzoli et al., 2019). With respect to the conference paper, we have detailed the Methods section to allow replication studies and have included experts' evaluations of violinists' performance in the dataset.

## Data Availability Statement

The raw data supporting the conclusions of this article will be made available by the authors, without undue reservation.

## Ethics Statement

The study involving human participants was reviewed and approved by the Ethics Committee of the University of Montreal (17-018-CERES-D). The patients/participants provided their written informed consent to participate in this study.

## Author Contributions

AT, BM, MB, and SD contributed to conception and design of the study. BM, EG, MB, and SD contributed to the acquisition and analysis or interpretation of data for the work. AT performed the statistical analysis and wrote the first draft of the manuscript. All authors contributed to manuscript revision, read, and approved the submitted version.

## Conflict of Interest

EG was employed by Wilder & Davis. The remaining authors declare that the research was conducted in the absence of any commercial or financial relationships that could be construed as a potential conflict of interest.

## Publisher's Note

All claims expressed in this article are solely those of the authors and do not necessarily represent those of their affiliated organizations, or those of the publisher, the editors and the reviewers. Any product that may be evaluated in this article, or claim that may be made by its manufacturer, is not guaranteed or endorsed by the publisher.
