# Supplementary material for: Effect of Bow Camber and Mass Distribution on Violinists' Preferences and Performance
Source: Front Psychol. 2021 Nov 3;12:769831. doi: 10.3389/fpsyg.2021.769831 (PMC8595599; doi:10.3389/fpsyg.2021.769831)
Supplement: Supplementary file 1 [file Table_1.DOCX]

**Supplementary material:** the bow modification process

*
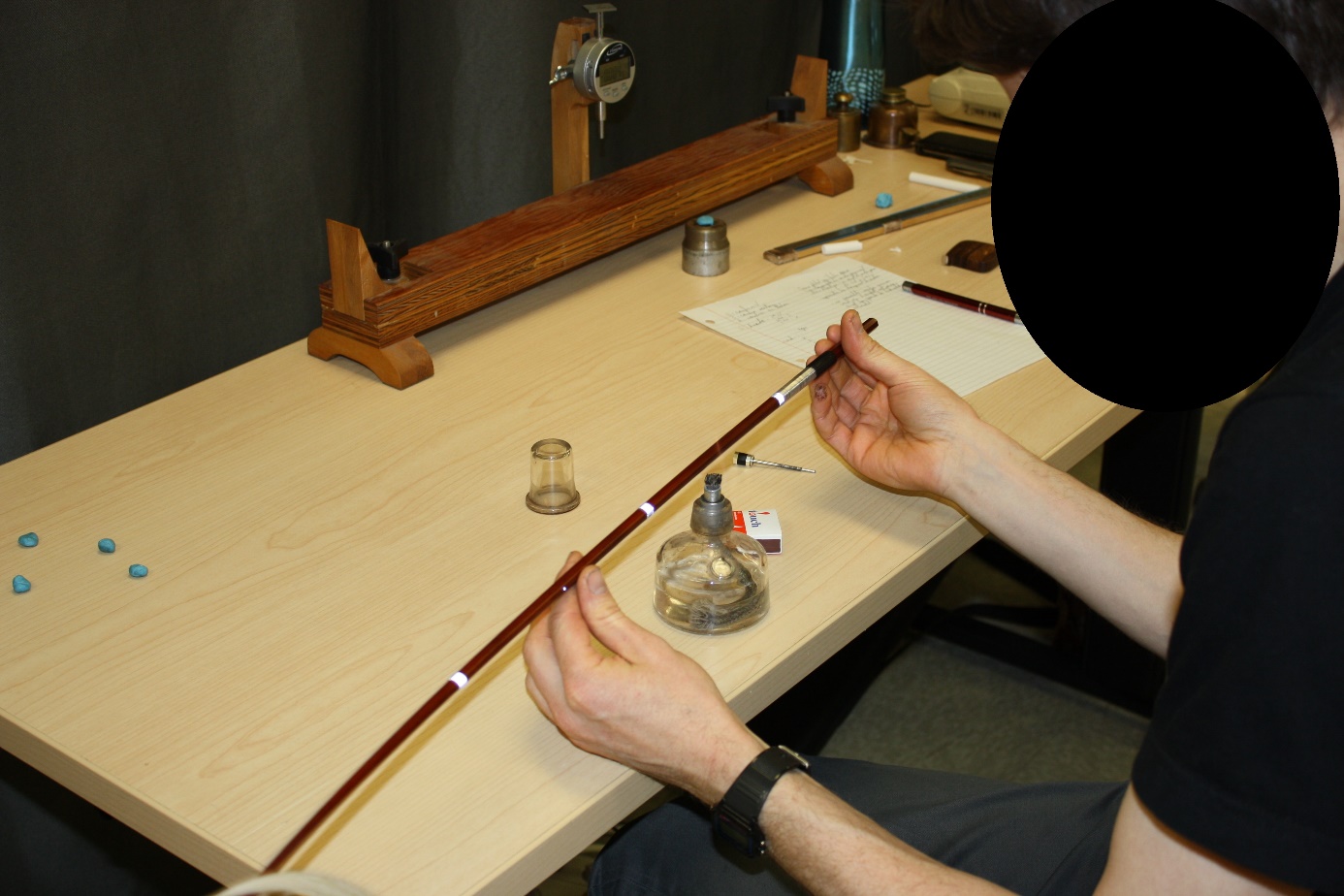
*

**Supplementary Figure 1.** The bow maker is heating the bow (red arrow in the middle of the picture) before changing its camber. Then, the blue gums (blue arrow on the left side of the pictures) are stuck to the bow tip and/or frog to adjust the bow mass.


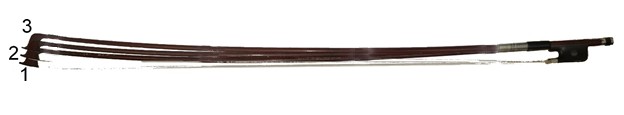


**Supplementary Figure 2.** The three bow cambers (1: maximal curvature near the bow tip, 2: at the middle, 3: near the frog).


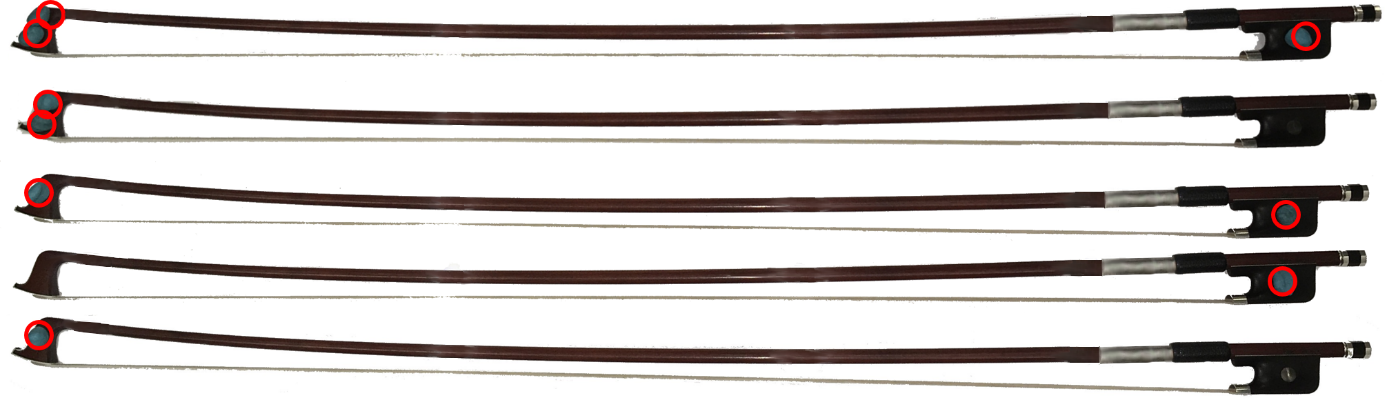


**Supplementary Figure 3.** Locations of the added masses (red circles).
